# Shell Model Reconstruction of Thin-Walled Structures from Point Clouds for Finite Element Modelling of Existing Steel Bridges

**DOI:** 10.3390/s25134167

**Published:** 2025-07-04

**Authors:** Tomoya Nakamizo, Mayuko Nishio

**Affiliations:** 1 Department of Engineering Mechanics and Energy, University of Tsukuba, 1-1-1 Tennodai, Tsukuba 3058573, Japan; 2 Institute of System and Information Engineering, University of Tsukuba, 1-1-1 Tennodai, Tsukuba 3058573, Japan; nishio@kz.tsukuba.ac.jp

**Keywords:** point cloud data, finite element model, shell element, steel girder bridge, quality assessment

## Abstract

Digital twin models utilising point cloud data have received significant attention for efficient bridge maintenance and performance assessment. There are some studies that show finite element (FE) models from point cloud data. While most of those approaches focus on modelling by solid elements, modelling of some civil structures, such as bridges, requires various uses of beam and shell elements. This study proposes a systematic approach for constructing shell element FE models from point cloud data of thin-walled structural members. The proposed methodology involves k-means clustering for point cloud segmentation into individual plates, principal component analysis for neutral plane estimation, and edge detection based on normal vector variations for geometric structure determination. Validation experiments using point cloud data of a steel corner specimen revealed dimensional errors up to 5 mm and angular errors up to 6°, but static load analysis demonstrated good accuracy with maximum displacement errors within 3.8% and maximum stress errors within 7.7% compared to nominal models. Additionally, the influence of point cloud data quality on FE model geometry and analysis results was evaluated based on geometric accuracy and point cloud density metrics, revealing that significant variations in density within the same surface lead to reduced neutral plane estimation accuracy. Furthermore, toward practical application to actual bridge structures, on-site measurements and quality evaluation of point cloud data from a steel plate girder bridge were conducted. The results showed that thickness errors in the bridge data reached up to 2 mm, while surface deviation RMSE ranged from 3 to 5 mm. This research contributes to establishing practical FE modelling procedures from point cloud data and providing a model validation framework that ensures appropriate abstraction in structural analysis.

## 1. Introduction

The bridge performance is quantitatively assessed to prioritise decisions regarding repair and reinforcement. This is achieved by constructing finite element (FE) models from drawings and design calculation sheets and performing structural analysis. However, constructing FE models for large-scale structures with complex geometries configured by assembling a lot of members, such as steel bridges, requires significant effort. Furthermore, the original drawings are sometimes unavailable. Recent efforts have focused on using optical measuring instruments, such as cameras and lasers, to obtain point cloud data of structures, aiming to enhance operational efficiency through digital twin model construction. Digital twins, in this study, refer to the process of constructing physical models in digital space from geometric information acquired from the real world and executing numerical simulations for structural analysis. Point cloud data consist of points with spatial coordinates, colour, and reflection intensity, serving as a 3D digital representation of the measured object. The ability to easily obtain 3D geometry from point clouds has led to research on reconstructing FE models of existing structures.

In general, FE models of bridges are constructed by selecting appropriate structural elements for structural members, based on the required displacement behaviours to resist design loads and the demand response outputs for evaluations [1]. Plate-like members, such as main girders, are modelled using shell elements, bar-like members, such as inclined braces, are represented with beam elements, and solid elements are employed for continuous bodies. By modelling each component separately, the mechanical behaviour based on member shapes is accurately represented, enabling an overall performance assessment of the bridge system, even when deterioration or damage occurs in a particular component.

In previous studies on the construction of FE models from point cloud data, many of target structures were historical masonry structures [2,3,4,5]. In those structures, it was reasonable to select the solid element, and those studies adopted methodologies: the surface mesh reconstruction, the cross-section stacking method, and the learning-based methods. The first approach to constructing a model involves directly using the surface point clouds of the target structure as nodes of the mesh [6]. For instance, Bassier et al. [7] employed the Poisson surface reconstruction method [8] to create a surface mesh from point clouds of masonry walls, converting it into a solid element FE model through mesh water tightening. This approach enabled the construction of an FE model that accurately captured structural details, such as volume reduction caused by deterioration. Similarly, Zhang et al. [9] developed surface meshes of damaged concrete sections and updated FE models by incorporating these damaged portions into existing models. This method demonstrated the feasibility of incorporating complex features, such as damage, into FE models. However, the surface mesh reconstruction methods, while capable of accurately capturing structural details, require handling point cloud data directly in three dimensions, leading to high computational costs depending on the number of points. Moreover, they are highly sensitive to the accuracy of point cloud data, leading to challenges such as the reconstruction of uneven surfaces due to outliers or surface variability, as well as the formation of holes in areas where meshes cannot be generated.

The second approach, the cross-section stacking, as demonstrated by Xu et al. [10] and Wang et al. [11], involved treating the structure as a stack of cross-sections, performing meshing processes for each cross-section, and then connecting them to create an overall model. Castellazzi et al. [12] used convex hull algorithms to obtain polygons outlining the contours of each cross-section of a stone statue and constructed solid element FE models by stacking these polygons. Zhang et al. [13] sliced the point cloud data of reinforced concrete (RC) beams along the axial direction and converted the 3D models of each slice into FE models using plane fitting with the least squares method. This method estimates surface shapes from point cloud data, enhancing robustness against errors and noise. However, the slicing approach presents challenges related to surface continuity, such as difficulties in smoothly connecting slices depending on the slicing width resolution, making the determination of an appropriate slicing width a critical consideration. Additionally, this method may not be directly applicable to objects with complex geometries. For instance, in steel structures where members have multiple faces oriented in various directions, slicing along a single axial direction is not feasible.

The third approach, the learning-based method, has emerged as a promising direction in recent years. For instance, Kim et al. [14] employed deep learning for segmenting scaffold point clouds and constructing 3D models of each segment. Additionally, Mahmoud et al. [15] presented a deep-learning-based framework for automatic reconstruction of 3D models of indoor point clouds, including furniture such as tables and chairs. However, those existing studies have primarily focused on segmentation tasks of acquired point cloud data [16,17,18] while deep learning models capable of directly reconstructing FE models suitable for structural analysis remain largely unexplored. This limitation comes from several challenges: inadequate availability of annotated datasets, difficulty in handling complex geometric structures, and the specific requirements of structural analysis that demands high precision in geometric properties such as material boundaries and load-bearing capacities.

Bridge structures, which consist of various structural members, require FE modelling that adopts multiple element types; not only the solid element but also the beam and shell elements. In studies utilising beam elements, Otero et al. [19] estimated frame structures and conducted structural analyses of a wooden roof, while Rúa et al. [20] computed the convex hull of I-beam cross-sections and constructed beam element FE models by calculating geometric properties such as cross-sectional area. A comparison with FE models using solid elements revealed a significant reduction in computational cost while maintaining sufficient accuracy in the numerical analysis results. Similarly, Smith and Sarlo [21] extracted centroid lines of I-beam sections by converting the sections into images. In studies utilising shell elements, Cui et al. [22] constructed element nodes by downsampling the point cloud data of tunnel interiors for each cross-section. Xu et al. [23] constructed a shell model by detecting edge curves of cross-sections from a deformed I-column. While these studies effectively employed slicing approaches, they did not fully address the previously mentioned challenges. Moreover, few studies have verified FE modelling using shell elements for thin-walled steel structures such as steel bridges. In the FE analysis of steel structures, shell elements are widely used to model various components such as main girders, slabs, and other thin-plate members. This highlights the need for further investigations into shell element FE modelling from point cloud data to enhance its applicability and accuracy for steel structures.

Additionally, the influence of data qualities of point clouds, such as non-uniform density, surface deviation, and missing parts, on FE model reconstruction should also be considered. Many previous studies indicated that data errors cause inaccurate model geometries. Geometric errors in the constructed model may lead to unreliable numerical analysis results. Previous studies have explored the relationship between point cloud data quality and the accuracy of constructed 3D models. Yu et al. [24] utilised geometric shapes, such as 3D planes estimated from point cloud data, to investigate construction quality, including building dimensions and flatness. Yan and Hajjar [25] demonstrated that variations in the number of scans significantly affected beam thickness dimensions. Anil et al. [26] analysed geometric deviation patterns, while Truong-Hong et al. [27] examined quality factors such as registration errors and outliers. Mohammadi et al. [28] and Rebolj et al. [29] introduced various indicators such as density, surface coverage, and depth accuracy to quantitatively represent point cloud data quality. However, these studies did not address the relationship between point cloud data qualities and constructed FE models, nor did they specify the level of point cloud accuracy required for practical applications.

This study aimed to establish a systematic procedure for constructing shell element FE models from the point cloud data of thin-walled structural members. It provides a quantitative evaluation of the influence of point cloud data quality on model accuracy and introduces a new perspective on the model validation process to ensure an appropriate level of abstraction in numerical analysis. This paper presents a novel systematic procedure that enables the separation of point cloud data from thin-walled structural members into individual flat plate components and the estimation of neutral planes and geometric structures required for shell model construction. The approach can be applied to complex structures where members are oriented in multiple directions, enabling appropriate FE modelling by assigning suitable structural elements according to the mechanical role of each member. The validity of the constructed models is evaluated through numerical analysis, while quantitative evaluation of point cloud data quality reveals its impact on the model construction process. While shell element models represent an abstracted form of three-dimensional structures, this study contributes not only to establishing a practical FE modelling procedure for steel structures from point cloud data but also to providing a comprehensive framework for model validation that ensures reasonable abstraction in structural analysis.

In the following sections, Section 2 describes the modelling method from point cloud data of an intact steel corner specimen, designed to represent the basic configuration of actual steel bridges, and discusses the validity of the modelling approach based on the construction results and data quality evaluation. Section 3 evaluates the point cloud data acquisition and quality of an actual steel bridge with the intent of applying the proposed modelling method to real bridge structures and examines quality attributes that may affect modelling. Finally, Section 4 summarises the research conclusions and future works.

## 2. Shell Model Reconstruction from Point Clouds of Thin-Walled Steel Members

This section presents the proposed method for constructing a shell element FE model from point cloud data, which involves segmenting each plate member and estimating the neutral plane. The method is validated using a geometry consisting of plates oriented in three orthogonal directions. This experimental configuration was determined based on the recognition that most steel structures are basically constructed by jointing rectangular plate members in multiple normal directions (predominantly orthogonal arrangements). Elastic analysis is then performed on the constructed FE model under a static loading to verify the validity of the model from output responses. Here, the main processes of model reconstruction were implemented by coding algorithms by Python 3.10 with libraries of Open3D 0.17 [30], and CloudCompare 2.12 [31], the open-source processing software, was used for some simple processing, such as visualisation and manual denoising.

### 2.1. Data Acquisition and Preprocessing

**Figure 1:**
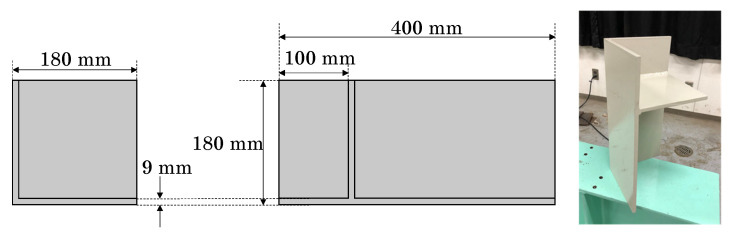
Drawing and photograph of steel corner specimen.

Most steel structures are assembled using steel plates configured at right angles. For basic verification, a steel corner specimen with a generalised geometry was prepared, as shown in Figure 1. The specimen consisted of three plates oriented along a rectangular coordinate system. The steel plates were 9 mm thick. Two main plates (180 mm × 400 mm) were welded at right angles to form a corner, and a square middle plate (171 mm × 171 mm) was welded 100 mm from the top.

The point cloud data of the steel corner specimen were acquired using a handheld 3D scanner, DPI-8X [32], in the laboratory. This device offers a measurement range of 0.3 m to 1.9 m, with a standard accuracy of ±2 mm at a reference distance of 1 m. Additionally, it provides a resolution of 1.7 mm at a distance of 1 m. Since it uses a structured light system, the sensor naturally produces rounded edges, and the thicker parts of the plate, which lack clear geometric features, are often measured less accurately in the point cloud data.

For data acquisition, the specimen was placed on a frame in the laboratory, and measurements were taken by slowly moving around the frame while holding the sensor. The point clouds were acquired three times, labelled Data #1 to #3, to assess variability between the datasets in terms of FE analysis results and quality. Each point cloud data point is defined as $P_{i}={\left[x_{i},y_{i},z_{i},R_{i},G_{i},B_{i},I_{i},\right]}^{T}(i=1,\ldots ,N)$, consisting of 3D coordinate values, RGB colour components, and reflection intensity. After data acquisition, CloudCompare was used to manually remove the background and extract the steel members. No point cloud data were acquired at the bottom of the specimen where it was in contact with the frame.

**Figure 2:**
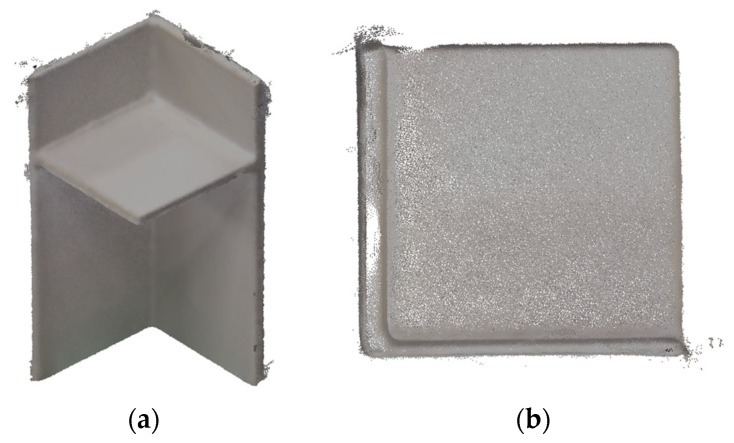
Raw point cloud of specimen before preprocessing (Data #2): (**a**) Whole View; (**b**) View of the upper face.

Figure 2 shows the raw point cloud data for Data #2. While the overall shape of the specimen is clearly captured, noise is noticeable near the edges. The external edges and corner points are typically rounded, with visible irregularities on the surfaces. Additionally, gaps were observed in the thickness sections where points were missing, and a significant variation in thickness across the members was evident.

Raw measured point cloud data typically contain a large number of points and exhibit noise, including outliers deviating from the target shape, as well as non-uniform point cloud density. To reduce computational costs and enable effective applications, preprocessing is necessary. This involves downsampling with density homogenisation and denoising processes. In this study, a voxel downsampling method was employed. This method involves placing a grid of cubes, called voxels, in space, calculating the centroid of the points within each voxel, and replacing them with a single point. This process reduces the number of points while maintaining nearly constant spacing between them, resulting in a point cloud with an overall uniform density. The key parameter in voxel downsampling is the voxel size. After testing sizes ranging from 1 to 4 mm and considering factors such as exceeding the scanner’s resolution and retaining points in small areas (e.g., plate thicknesses), a voxel size of 2 mm was selected.

For noise removal, a statistical outlier removal method [33] was used. This method detects and eliminates outliers based on the average and standard deviation of the distances between points. For each point $P_{i}$, the average distance $\mu_{i}$ to all neighbouring points is computed. Assuming the resulting distribution follows a Gaussian distribution with mean $\hat{\mu }$ and standard deviation σ, points lying outside the interval defined by $\hat{\mu }+n_{STD}\cdot \sigma$ are considered outliers and removed. Here, $n_{STD}$ represents the standard deviation multiplier. Key parameters for this method include the number of neighbouring points $N_{neighbor}$ used to compute the average distance $\mu_{i}$ and the standard deviation multiplier $n_{STD}$. If these parameters are too small, normal points may be misclassified as outliers, whereas excessively large parameters may fail to remove true outliers. Through trial and error during validation, $N_{neighbor}=10$ and $n_{STD}=3.0$ were selected.

Furthermore, the normal vectors of the points were calculated for use in reconstructing the shell model geometry, as discussed in the next section. A normal vector indicates the direction of the plane in which the point lies. By performing principal component analysis (PCA) on the local neighbourhood of each target point, the local plane is estimated, and its normal direction, known as the normal vector, is determined. PCA is a technique that transforms data into a new coordinate system where the variance is maximised and is commonly used for dimensionality reduction.

The local plane estimated from the point cloud is spanned by the axes of the first and second principal components $u_{1}$ and $u_{2}$, while the third principal component $u_{3}$, which is orthogonal to them, represents the direction of the normal vector. The parameters used for the PCA calculation included the calculation radius r from the target point and the maximum number of points $N_{max}$ used in the calculation. For this validation, r=4.0 mm and $N_{max}=20$ were selected based on a voxel size of 2 mm and a plate thickness of 9 mm, ensuring accurate normal calculation even in thicker areas of the plate.

The preprocessing results are summarised in Table 1, which includes the number of points before and after preprocessing, along with the number and percentage of outliers. Although the initial number of points varied significantly, ranging from approximately 1 to 4.5 million points depending on the dataset, the number was reduced to approximately 150,000 points through downsampling.

### 2.2. Shell Model Geometry Reconstruction

The shell element model geometry was constructed by assembling the representative planes and neutral planes of the steel corner specimen. Two essential procedures—estimation of the neutral planes and recognition of the dimensions of each plane—are detailed, and the accuracies and variations of the constructed shell geometries are evaluated.

#### 2.2.1. Estimation of Neutral Planes of Plates

The neutral planes of the steel plate configurations were estimated to construct the geometric structure of the shell model from the point clouds. These processes were implemented using the scikit-learn library [34]. Initially, the preprocessed point clouds were segmented into individual flat plate components using the k-means clustering method. K-means clustering groups data points into a specified number of clusters based on their similarity. Initially, k centroids, $\mu_{j}\;\left(j=1,\ldots ,k\right)$, are arbitrarily placed in space, and each point $x_{i}={\left[x_{i},y_{i},z_{i},n_{xi},n_{yi},n_{zi}\right]}^{T}$ is assigned to the nearest centroid to form clusters $C_{j}$. Note that ${\left[n_{xi},n_{yi},n_{zi}\right]}^{T}$ is the normal vector component value of each point. Since the normal direction is useful information in the clustering of corner members, it was employed as a feature vector. New centroids are then calculated from the point clouds of each cluster, and the process of assigning each point to the nearest centroid is repeated. This iterative algorithm minimises the objective function, described by Equation (1). Clustering was completed when the centroids no longer updated, allowing the specimens to be successfully segmented into individual flat members.

(1) $$J=\sum_{i=1}^{N}\sum_{j=1}^{k}{\left\|x_{i}-\mu_{j}\right\|}^{2}$$

**Figure 3:**
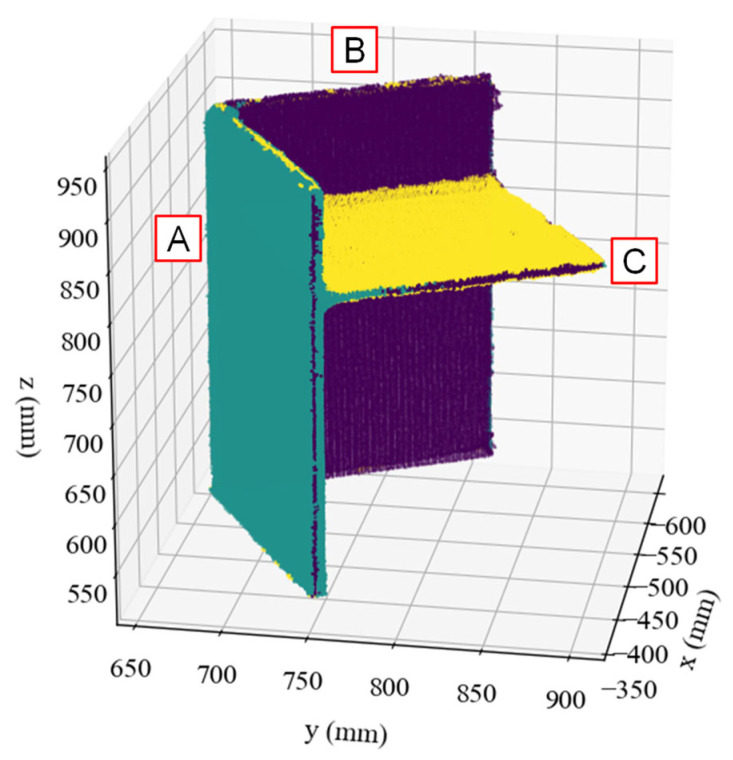
Segmentation result of Data #1. Different colors indicate different segmented components. Labels A, B, and C denote segmented plate members.

Figure 3 shows the segmentation results of Data #1, where the three clusters are represented by different colours in the original coordinate system. Using the normal vector components as feature vectors enabled successful segmentation of each flat plate. However, the points corresponding to the plate thickness were classified into the same cluster as those of the corresponding flat plate. Similar segmentation results were observed for the other datasets.

To estimate the neutral planes for each segmented plate, the following procedures were implemented. First, point clouds corresponding to the plate thickness, which could affect the accuracy of plane estimation, were removed. This was achieved using the outlier removal algorithm described in the previous section, applied to each plate’s point cloud with $N_{neighbor}=180$ and $n_{STD}=0.4$. These parameter values were determined through a trial-and-error process to effectively remove as much point cloud data associated with the plate thickness as possible.

After the outlier removal, PCA was applied to the remaining point clouds of each plate. In PCA, the eigenvectors $u_{1}$ and $u_{2}$, corresponding to the two largest eigenvalues, represent the in-plane directions of the plate, while the eigenvector $u_{3}$, corresponding to the smallest eigenvalue, represents the normal vector of the neutral plane. Using these principal components, the point clouds were transformed into local plate coordinate systems ($u_{1},u_{2},u_{3}$) for each plate.

**Figure 4:**
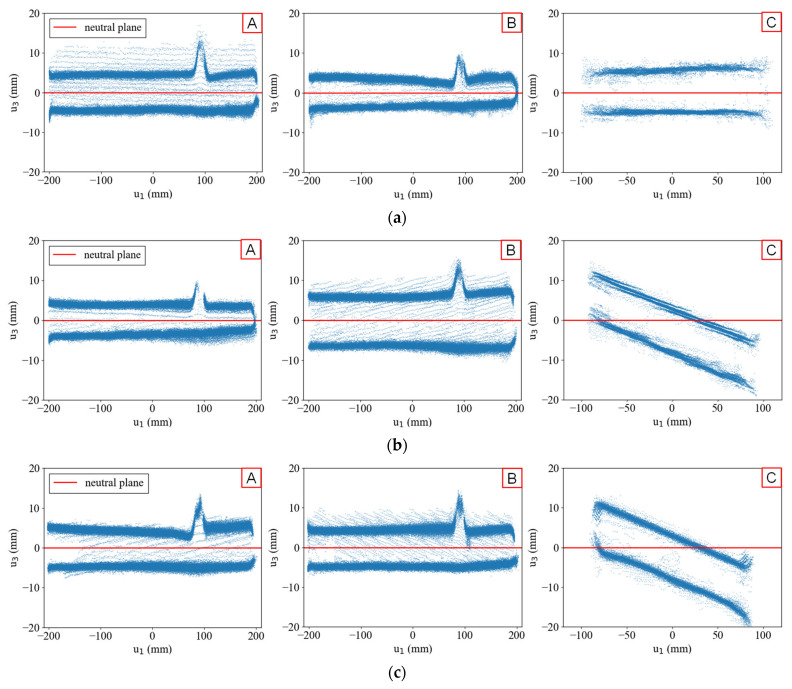
Neutral plane estimation results: (**a**) Data #1; (**b**) Data #2; (**c**) Data #3. Labels A to C indicate plates accordance with Figure 3. Blue dots are point cloud data, and red line is estimated neutral plane, in each figure.

Figure 4 shows the point clouds transformed into their respective local plate coordinate systems, with the red lines indicating the neutral planes at the centroid of each point cloud. While the estimated neutral planes generally aligned well with most plates, the data exhibited two issues: surface distortion of thin-walled members and incomplete segmentation at the joint sections. Surface distortion appears as point scatter along the $u_{3}$ direction, whereas incomplete segmentation is evident from the remaining points near the joints, which could not be clearly separated during the clustering process. Notably, the neutral planes of Plate C in Data #2 and #3 deviate from their expected normal directions.

To quantitatively analyse these characteristics, the point cloud density was calculated by counting the points within a 3 mm radius sphere centred on each point. This radius was chosen based on the 9 mm plate thickness to ensure that only coplanar neighbouring points were included. Generally, point cloud density increases as the measurement device approaches the object and decreases with distance or in areas that are challenging to measure.

**Figure 5:**
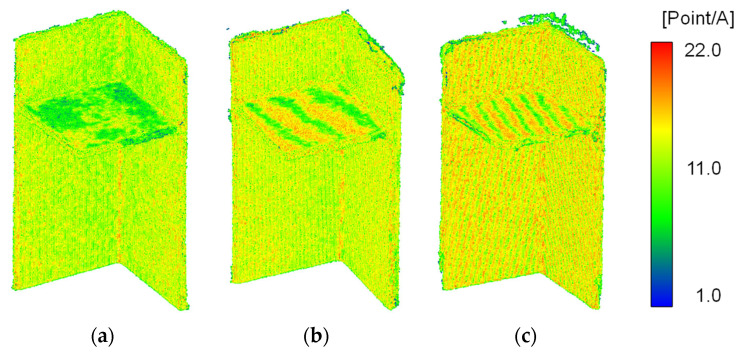
Point clouds density ($A=36\pi \;{mm}^{3}$): (**a**) Data #1; (**b**) Data #2; (**c**) Data #3.

Figure 5 highlights areas of lower point cloud density, specifically along edges with noise and in thicker plate regions with data gaps. Differences in the number of points between the two surfaces of the flat plate were also observed. Data #2 and #3 exhibited distinctive stripe-like density variations within individual plates, attributed to voxel downsampling during preprocessing. Unlike Data #1, the initial measurement coordinate systems for Data #2 and #3 were misaligned with the flat plate normal directions. Consequently, when the downsampling grid intersected the flat surfaces diagonally, stripe patterns were introduced, which affected the accuracy of neutral plane estimation.

Since PCA optimises the variance of the data along its axes, these density variations can negatively impact the accuracy of geometric estimation, particularly in the calculation of angles between members. These findings underscore the need for two preprocessing improvements: (1) standardising the coordinate system before analysis and (2) refining downsampling methods to ensure a uniform density distribution.

#### 2.2.2. Edge Extraction for Determining Dimensions

**Figure 6:**
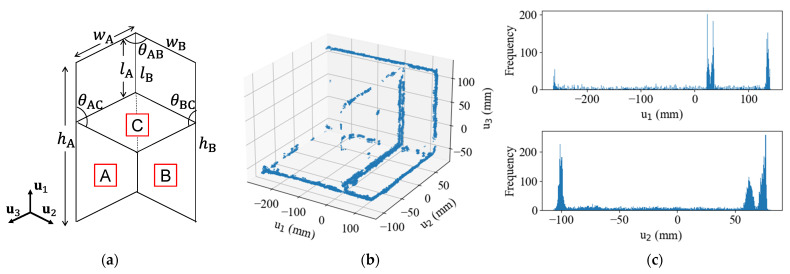
(**a**) Names of dimensions; (**b**) Edge point clouds of Data #1; (**c**) Histograms for each coordinate axis. Labels A, B, and C indicate plates accordance with Figure 3. The dimensions of the shell model are listed in Table 2. The intersection angles between the plates showed errors of less than 1°, except for Data #2 and Data #3, which exhibited deviations of approximately 6°. As discussed in the previous section, Data #2 and Data #3 display variations in density distribution within the flat plate members. The measured values of $\bar{h}$ and w were consistently smaller than the design specifications, with maximum deviations of 4.95 mm. The measured $\bar{l}$ values showed bidirectional deviations: they were larger than the design values in Data #1 and #2, but smaller in Data #3.

Figure 6a illustrates the primary geometric dimensions of the specimen, including the height h, width w, length l from the upper end to the intermediate plate, and the intersection angle θ of each plate. The design values, accounting for plate thickness, are h=400, w=175.5, l=104.5 mm, and θ=90°.

Geometric estimation was conducted in two sequential steps: in the first step, the intersection angle θ between each pair of plates was calculated using the dot product of the normal vectors of their respective neutral planes. The second step involved detecting edges based on variations in the normal vectors between points to estimate the geometric structure. As a point approaches an edge, its normal vector gradually shifts toward the direction perpendicular to the adjacent surface. Using this characteristic, edges were detected by setting an angle threshold γ on normal vector changes. For each target point$\;x_{i}$, the dot product α was calculated between the normal vector $n_{i}$ of the target point and the normal vectors $n_{j}\;(j=1,\ldots ,N_{k})$ of neighbouring points $N_{k}$. The number of neighbouring points $N_{k}\;$was set to 15, and the angel threshold γ was chosen as 0.643, corresponding to an angle of 50.0 degrees between $n_{i}$ and $n_{j}$. These parameters were determined through systematic testing, evaluating angles from 40° to 80° in 10-degree increments and neighbouring points from 5 to 20 in steps of 5.

Considering the influence of point cloud data quality discussed in the previous subsection, the remaining dimensions were determined by statistically processing the edge point cloud. The edge point clouds were transformed into a local plate coordinate system defined by the principal components $\left(u_{1},u_{2},u_{3}\right)$ of Plates A and B. Coordinate value histograms were created for dimensional estimation, with a histogram bin width of 0.5 mm to ensure sufficient precision for measurements. For example, Figure 6b shows the edge point clouds of Data #1 transformed into the local plate coordinate system of Plate B, along with the histograms for each coordinate axis, which are shown in Figure 6c. Peaks along the $u_{1}$-axis corresponded to the bottom end, upper end, and the junction with intermediate Plate C, while peaks along the $u_{2}$-axis indicated the long edges of Plate B and the junction between Plates A and B. To determine the dimensions h, l, and w, peak thresholds (e.g., 40 for Data #1) were applied to the histograms to identify the positions of edges and neutral planes. The distances between the centroids of the extracted peaks, representing identified positions, were then calculated.

Because the number of edge points varied across datasets, the threshold was adjusted for each dataset to ensure accurate peak detection. The final dimensional values were derived from the mean $\bar{h}\;$and $\bar{l}$ values obtained from measurements of Plates A and B.

A limitation of each processing step is that parameter settings are not fully automated, because the total number of points and noise distribution vary across datasets. Among the processing steps, edge detection requires the highest computational and manual effort costs. While quantitative computational cost evaluation has not yet been conducted, edge detection is likely to become a bottleneck in automation due to its requirement for subjective parameter tuning. To address this challenge for improved modelling accuracy with process automation, parameter optimisation is necessary. This can be addressed through parameter sensitivity analysis as well as utilising quantitative evaluation metrics such as point cloud density.

### 2.3. Validation of FE Model by Using Constructed Shell Element Geometries

A numerical analysis was conducted to derive the physical quantities of the demand outputs, such as responses to external loads, based on the purpose of the simulation. From the perspective of model validation, the reliability of the numerical model depends on the accuracy of the demand outputs. For instance, in the performance analysis of a bridge, global responses, such as deflection under live loads and maximum displacements during earthquake loads, are evaluated to meet specific performance requirements. Conversely, local responses, such as localised stresses in structural elements, are assessed for other performance criteria. The required quality and fidelity of FE models vary depending on the specific demand outputs, highlighting that not all outputs necessitate the same level of precision.

In this study, the validity of the FE model constructed from point cloud data (the “PC-FE model”) was verified by comparing its responses under static loading to those of the nominal FE model, which was constructed using the designed dimensions. FE analyses were performed using the general-purpose FE analysis software Abaqus 2022. The geometry of the shell model was created based on the dimensions determined from the point cloud data, as presented in Table 2. A nominal plate thickness of 9 mm, considered known information, was assigned. The steel material properties used for the specimen included a Young’s modulus of 205 GPa and a Poisson’s ratio of 0.3 for elastic calculations. The shell FE model employed a four-node quadrilateral first-order element with a mesh size of 3 mm to ensure optimal convergence of the output.

**Figure 7:**
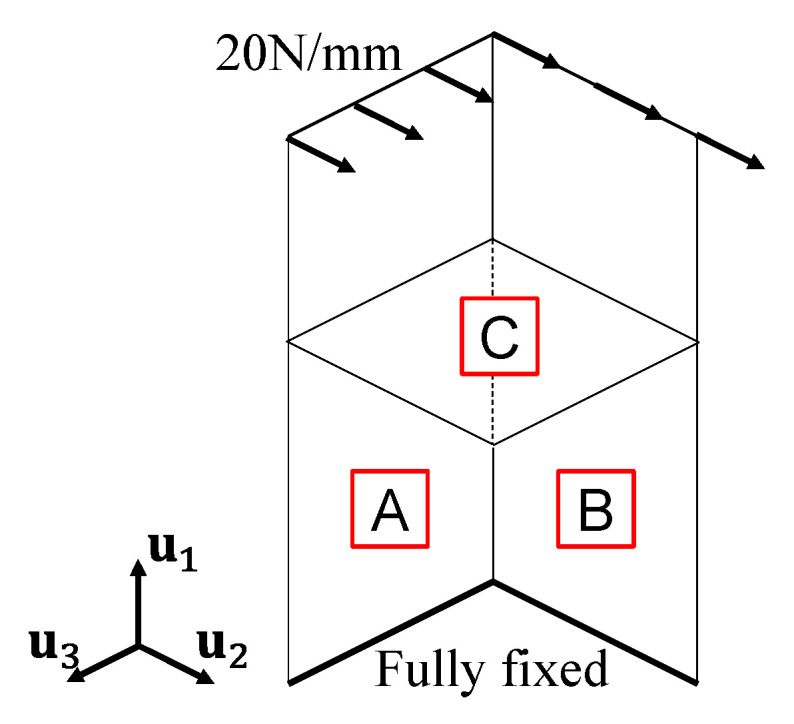
Boundary and loading conditions. Labels A to C are defined in accordance with Figure 3.

Boundary conditions were defined by fully constraining all six degrees of freedom at the lower ends of the member, as illustrated in Figure 7. A distributed load was applied along the top edge of Plates A, B in the direction normal to the plate. The total load magnitude was 7020 N, uniformly distributed along the upper edge length, which varied among the PC-FE models due to geometric differences. In the nominal model, this load corresponded to 20 N/mm. This loading configuration induced deep beam-bending behaviour in the overall structure. At the component level, each L-shaped section exhibited combined bending and shear deformation, while Plate C underwent torsional deformation due to its connection with adjacent members. The evaluation focused on both global responses, such as the von Mises stress distribution and maximum displacement $u_{max}$, and local responses, including the maximum von Mises stress $\sigma_{max}$.

**Figure 8:**
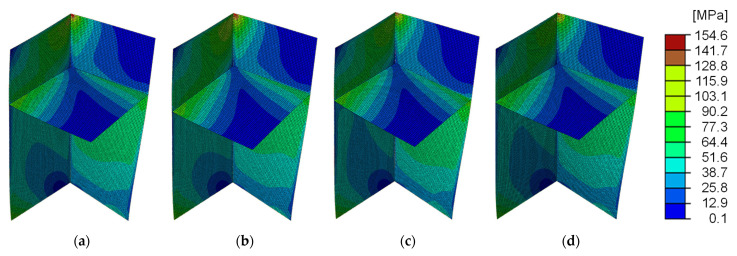
Von Mises stress distribution of point cloud FE model: (**a**) Data #1; (**b**) Data #2; (**c**) Data #3; (**d**) Nominal model.

Table 3 compares the output responses between the PC-FE models and the nominal models, while Figure 8 illustrates the overall von Mises stress distribution. The maximum displacement $u_{max}$, which is crucial for understanding overall model behaviour, was predominantly observed at the upper left end of the specimen across all cases. The results were generally favourable, with a maximum relative error of 3.8% in Data #2. The stress distributions were generally consistent, with minimal variation across datasets.

The maximum von Mises stress $\sigma_{max}$, essential for assessing stress concentration within the structure, was consistently observed at the upper-end joint of the specimen in all cases. While the results were favourable overall, Data #2 exhibited a relative error of 7.7%. In Data #3, where both $u_{max}$ and $\sigma_{max}$ were smaller than the design values, the results in Table 2 suggest that the higher positioning of the intermediate plate increased stiffness against bending. Conversely, in the PC-FE model of Data #2, the anticipated angular error $\theta_{BC}$ resulted in both $u_{max}$ and $\sigma_{max}\;$showing larger values and relative errors compared to the design values, highlighting the angular error’s significant impact on the analysis outcomes. However, no significant degradation in the analysis results was observed due to $\theta_{AC}$ error in Data #3.

Overall, the analysis outputs of the PC-FE models demonstrated favourable results for both global and localised responses compared to the nominal models. Nevertheless, the accuracy of the geometric structure estimation, particularly in dimensions and angles, was shown to influence the analysis results.

## 3. Quality Evaluation of Bridge Point Cloud Acquired in On-Site Measurement

In the previous section, it was confirmed that inaccuracies in the dimensions and angles estimated from the point cloud data contributed to errors in the maximum stress and maximum displacement in the analysis results. Therefore, this section aims to evaluate the quality of point cloud data obtained from on-site measurements of a real bridge, with a view to applying the proposed shell element FE modelling method. The evaluation focuses on key quality factors, including geometric accuracy, surface deviation, and point cloud density, and discusses how these factors influence the reconstruction and accuracy of FE models.

### 3.1. Target Bridge and Data Acquisition

The target bridge is a single-span simply supported steel plate girder bridge with a length of 25 m and a width of 6.5 m, which has been in service since 1985. It is one of the spans of a ramp bridge connecting to a highway road and consists of concrete piers, concrete slabs, three main I-girders, cross beams, steel bearings, and other steel structural members in the superstructure.

**Figure 9:**
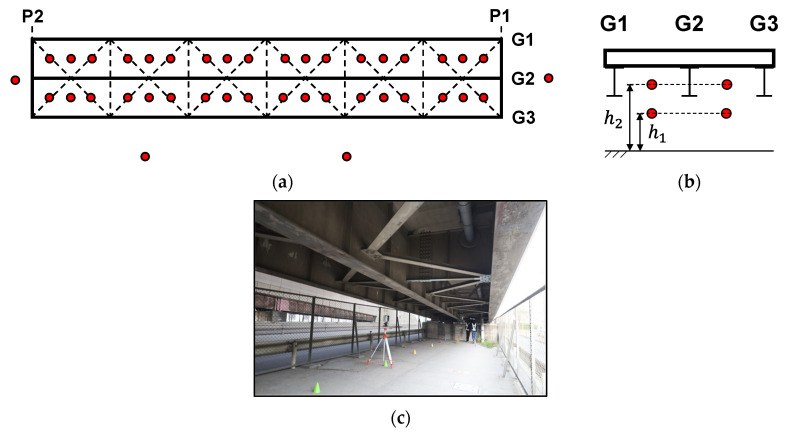
Details of measurement points: (**a**) Horizontal view; (**b**) Cross-sectional view; (**c**) Measurement setup under the bridge girder. P1 and P2 indicate the positions of the bridge piers, and G1, G2, and G3 represent the main girders. Red circles in (**a**) and (**b**) mark the measurement points. The dotted line in (**a**) indicates bridge members of cross beams and sway bracings.

Point cloud data were acquired using a terrestrial laser scanner (RTC360, Leica Geosystems). This scanner employs time-of-flight laser technology with a scanning range of 0.5–130 m, a coordinate accuracy of ±1.9 mm, and a resolution of 3.0 mm at a reflection distance of 10 m. To perform detailed surface measurements and capture point cloud data for the structural members across the entire bridge, multipoint scanning was conducted from 76 measurement locations. Of these, 72 points were located under the girders, and four were positioned around the bridge, as indicated by the red circles in Figure 9a–c.

The scanning points under the girders were arranged in two layers: one at a height of approximately 1.5 m *h*_1_, where the scanner was placed beneath the lower flange of the girders, and another at a height of 2.5 m *h*_2_, where the scanner was positioned above the lower flange and sway bracings, as shown in Figure 9b. The data acquired at each location were automatically synthesised using the real-time registration functionality of the laser scanner and the point cloud processing software Cyclone REGISTER 360 [35].

**Figure 10:**
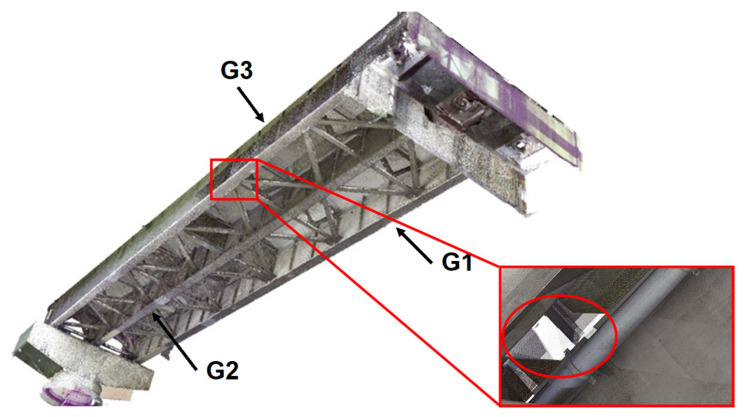
Acquired point cloud data around end-of-girder. G1, G2, and G3 indicates main girders accordance with Figure 9. A part with data missing is enlarged and highlighted, by red squares and a red circle.

Figure 10 shows the point cloud data obtained after preprocessing, including the manual removal of background objects. The dataset consists of over 1.3 billion points, with a total data size of approximately 20 GB in LAS format. Overall, the data were sufficiently clear to identify structural components at a detailed level, including primary members as well as secondary elements such as stiffeners and sway bracings. No significant defects attributable to the multipoint scanning process were observed. However, there were some typical missing parts, such as areas of the slab behind pipelines or other secondary members, especially near the tops of the horizontal stiffeners above $h_{2}$, and the exterior portion of the main girder G1 due to occlusions caused by neighbouring members. Despite these gaps, most surface plates and the thicknesses of the plate girders exhibited minimal missing points. Therefore, the normal vectors required for geometric estimation in FE modelling are expected to be appropriately derived. The minimal presence of missing points enhances the reliability of normal vector computation, thereby improving the precision of edge extraction and subsequent geometric reconstruction.

### 3.2. Quality Assessment of Acquired Bridge Point Clouds

To assess how the quality of point cloud data obtained from on-site measurements affects shell model reconstruction, three evaluation indices were defined: geometric accuracy, surface deviation, and point cloud density. These indices were calculated using CloudCompare, based on the methodology outlined in Section 2.

To evaluate the geometric accuracy, the member sections of the main girder G2, where measurements were possible from both sides, were analysed for the following cross-sectional dimensions: height $H_{w}$, web thickness $T_{w}$, bottom flange width $W_{bf}$, and bottom flange thickness $T_{bf}$. The evaluation process involved manually extracting the web and flange surfaces of G2 and dividing the surface point clouds for each plate member. The upper flange surface beneath the slab was treated as embedded in the slab, resulting in the extraction of seven surface point clouds: four from the bottom flange, two from the web, and one from the upper flange.

For each of these point clouds, plane fitting was performed using PCA, and the distances between the reference point cloud and a comparison mesh were calculated using the cloud-to-mesh (C2M) distance method. C2M measures the distance between a reference point cloud and a comparison mesh. By adjusting the combinations of the surface point clouds in the distance calculations, the dimensional values of interest were derived.

**Figure 11:**
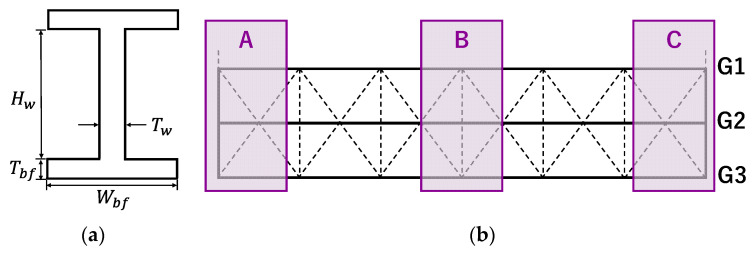
(**a**) Cross-sectional evaluation items and (**b**) target areas. Areas highlighted by purple squares with labels of A, B, and C, where are areas of midspan and both ends of girders, indicate regions selected for assessing qualities of the point clouds, covering the midspan and both ends of the girder.

This evaluation was conducted in three areas of the main girder G2, labelled A, B, and C, as shown in Figure 11: Area B: located at the centre of the girder, where the bending moment is critical due to live loads. Areas A and C: located near the ends of the girder, where localised damage such as corrosion or fatigue cracks often occurs. This damage can significantly affect the overall load-carrying capacity of the bridge.

Table 4 presents the evaluation results for the local dimensions of the main girder G2 section. The overall geometric errors were within a few millimetres, with a maximum error of 15.6% observed for $T_{w}$ in Area C. The relative error was more pronounced in thicker sections of the plate, where the design dimensions were smaller than those in other areas.

As discussed in Section 2.3, even small geometric errors of a few millimetres can lead to significant inaccuracies in the maximum displacement and von Mises stress values during analysis. Notably, the errors in plate thickness were captured with relatively higher accuracy compared to those of the test member, which exhibited a maximum error of 3 mm.

While point clouds on the surface of an object should ideally be acquired smoothly, random and trend noise due to various factors cannot be avoided in actual data. Such noise causes disturbances in the estimated normal vectors on the object surface and affects the derivation of structural geometries, as demonstrated in the previous experimental study using histograms and the PCA process. To evaluate the extent of these disturbances, the deviation of each point from the surface was calculated.

The root mean square error (*RMSE*) of the deviation of each point from the plane fitted to the target point clouds was used as an evaluation metric. PCA was employed to fit a plane, and the *RMSE* was calculated based on the deviation $D_{i}$ of each point $P_{i}\;\left(i=1,\ldots ,N\right)$ from the estimated plane. The *RMSE* was determined using the following equation:

(2) $$RMSE=\sqrt{\frac{1}{N}\sum_{i=1}^{N}{D_{i}}^{2}}$$

The evaluation area for surface deviation included the web surfaces of the main girders G1, G2, and G3, as well as the cross-beam CB. The evaluation was based on the *RMSE* of the deviations of each point from the planes fitted to these web surfaces. For G2, G3, and CB, where point clouds were available on both sides of the web, the fitted planes were treated as neutral planes. Considering a web thickness of 9 mm for the girders, the *RMSE* of the distances from each point to the web neutral plane ideally equates to 4.5 mm.

Table 5 presents the evaluation results for the surface deviations. Notably, G1, where fewer point clouds were obtained on the outside web surface, exhibited an *RMSE* of 3.00 mm. In contrast, G2, G3, and CB, where both web surfaces were measured, showed *RMSE* values ranging from 4 to 5 mm, with deviations from the neutral plane ranging from 0.15 to 0.46 mm.

The greater surface deviation observed in G1 could potentially impact the accuracy of dimension estimation. To address this, the influence of surface deviations can be mitigated by subdividing the point cloud data into smaller areas and applying localised modelling using a slice-based method.

Finally, the point cloud density was evaluated using a search sphere with a radius of 3 mm. Variations in density distribution can affect the accuracy of neutral plane estimation for flat members using PCA, which in turn impacts the geometric accuracy between structural members. Due to the computational complexity of calculating point cloud density for the entire bridge, the analysis was limited to Area B of girders G1, G2, and G3.

**Figure 12:**
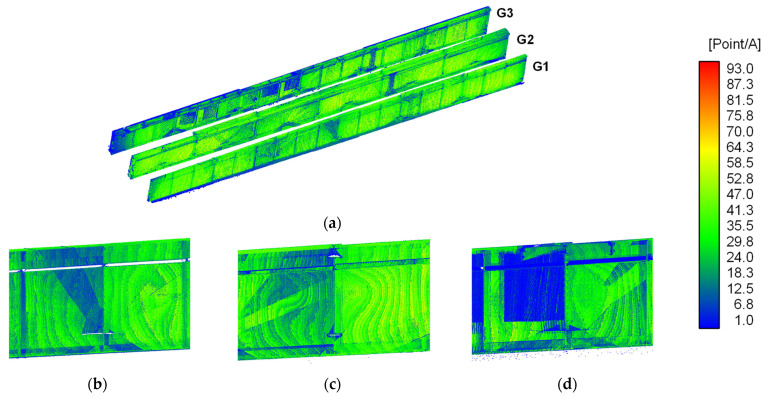
Point cloud density distribution of the plate girders ($A=36\pi \;{mm}^{3}$): (**a**) Whole plate girders; (**b**) G1 in Area B; (**c**) G2 in Area B; (**d**) G3 in Area B. G1, G2, and G3 are identifications of main girders accordance with Figure 9.

The results are shown in Figure 12. In general, the point cloud density on the web surfaces averaged approximately 40 points across most regions. However, localised reductions were observed at member joints, external edges, and around bolt locations. Moreover, geometrically patterned low-density regions were identified, likely caused by occlusion effects from specific viewpoints. Variations in point cloud density between the two surfaces of the girders were also detected, likely due to differences in the number of measurement points, with these disparities being more prominent in G3.

Based on these findings and the discussion in Section 2.2.1, accurate neutral plane estimation can be achieved by transforming the data from the initial coordinate system into a local system aligned with the normal direction of each flat surface of the main girders. This process can be further improved through the application of appropriate downsampling techniques. The low point density near the joints is expected to make the modelling difficult and affect the accuracy of the structural analysis. To address this issue, data acquisition with multiple devices and fusion with images can be considered [36].

## 4. Conclusions

This study proposed a systematic procedure for constructing shell element FE models from point cloud data of thin-walled structural members. The key contributions include establishing practical modelling techniques specifically designed for steel structures and developing a comprehensive validation framework that ensures reasonable abstraction in structural analysis. The main findings are summarised below.

- This research developed a comprehensive methodology combining k-means clustering for plate segmentation, PCA-based neutral plane estimation, and normal-vector-based edge detection to estimate the geometric structures of a steel corner specimen. The reconstructed geometry exhibited dimensional and angular errors of up to 5 mm and 6°, respectively. Point cloud density variations were identified as a key factor affecting model accuracy.
- The FE models constructed from the point cloud data were validated under static loading, demonstrating good agreement in the von Mises stress distribution compared to the nominal model. This validation demonstrated a quantitative relationship between point cloud data quality and FE model accuracy, with displacement errors within 3.8% and stress errors within 7.7%. These findings provide practical guidelines for determining acceptable data quality levels for structural analysis applications.
- Comprehensive in situ measurements using a ground-based laser scanner were conducted to apply the proposed shell element FE modelling technique to a real steel girder bridge. Despite occlusions in certain areas, the data provided a clear representation of the structural members, with only a few missing points.
- To apply the proposed modelling method to actual bridge structures, quality evaluation of point cloud data from a real steel girder bridge was conducted. The field measurements demonstrated relatively good geometric accuracy with dimensional errors ≤2 mm and surface deviation RMSE of 3–5 mm, while point cloud density averaged 40 points with localised reductions at joints and due to occlusion effects. These quality assessments provide critical data for future FE model construction of existing bridge structures.

These findings demonstrate that the quality of point cloud data significantly affects the accuracy of model reconstruction and analysis results. While the methodology is independent of acquisition methods, data quality varies between photogrammetry and active scanning, affecting validity. Therefore, examining the relationship between data quality and model accuracy provides significant insights applicable across different acquisition approaches. Furthermore, the systematic procedure provides a critical foundation for digital twin applications in bridge management by enabling accurate geometric modelling from point cloud data.

However, while this study focused on shell element modelling of a simple steel specimens, several important limitations and future research directions should be addressed. First, real bridge structures require various structural elements, including shell, solid, and beam elements, combined into comprehensive FE models, necessitating the development of integrated approaches to automatically construct these diverse element types from point cloud data. Additionally, advanced segmentation techniques using machine learning methods should be developed to automatically recognise various bridge components, including cross beams, stiffeners, and connection details. Moreover, a critical extension of this methodology involves addressing structures with damage, such as corrosion, cracks, and deformation, enabling assessment of damaged condition impact on structural performance. In this context, combining the proposed systematic procedure with damage detection techniques from point cloud data could enable modelling of structures with existing defects. It is also important that the quality assessments in Section 3 identified challenges such as localised density reductions at joints and occlusion effects that affect geometric estimation accuracy. Therefore, future research should develop robust preprocessing techniques as well as automated parameter optimisation to address these data quality issues, including noise reduction, missing data completion [37], and density normalisation methods [38], particularly for complex joints where point cloud quality is often compromised.

## Figures and Tables

**Table 1:** Data specifications.

	Data #1 *	Data #2	Data #3
Number of raw points	1,771,593	2,564,949	4,418,068
Number of points after preprocessing	150,243	157,207	165,054
Number of outlier points	194 (0.13%)	1523 (0.97%)	2087 (1.26%)

* “#” denotes “number”.

**Table 2:** Comparison of determined dimensions and intersection angles to nominal values.

	Data #1 *	Data #2	Data #3	Nominal Value
$\theta_{AB}$ [deg]	89.43 (−0.57)	89.27 (−0.73)	88.94 (−1.06)	90.00
$\theta_{AC}$ [deg]	89.95 (−0.05)	89.48 (−0.52)	95.68 (+5.68)	90.00
$\theta_{BC}$ [deg]	89.85 (−0.15)	95.91 (+5.91)	89.78 (−0.22)	90.00
$\bar{h}$ [mm]	396.07 (−3.93)	397.04 (−2.96)	398.17 (−1.83)	400.00
$w_{A}$ [mm]	171.41 (−3.59)	170.85 (−4.15)	171.05 (−3.95)	175.50
$w_{B}$ [mm]	170.10 (−4.90)	170.60 (−4.40)	170.05 (−4.95)	175.50
$\bar{l}$ [mm]	106.61 (+2.11)	106.29 (+1.79)	103.11 (−1.39)	104.50

* “#”: number (same as in Table 1).

**Table 3:** FE analysis results: maximum displacement and von Mises stress values.

	Model #1 *	Model #2	Model #3	Nominal Model
$u_{max}$ [mm]	2.31 (−0.16%)	2.40 (3.81%)	2.25 (−2.88%)	2.31
$\sigma_{max}$ [MPa]	147.03 (2.47%)	154.58 (7.74%)	142.16 (−0.92%)	146.30

* “#”: number (same as in Table 1).

**Table 4:** Evaluation of local dimensions of G2.

	Area	Mean [mm]	Design [mm]	Error [mm]	Error [%]
$H_{w}$	A	1890.12	1895	4.88	−0.26
	B	1717.55	1725	7.45	−0.43
	C	1551.42	1555	3.58	−0.23
$W_{bf}$	A	269.35	270	0.65	−0.24
	B	349.38	350	0.62	−0.18
	C	268.82	270	1.18	−0.44
$T_{bf}$	A	11.22	10	1.22	+12.20
	B	24.27	22	2.27	+10.32
	C	14.88	14	0.88	+6.29
$T_{w}$	A	9.45	9	0.45	+5.00
	B	8.71	9	0.29	−3.22
	C	7.60	9	1.40	−15.56

**Table 5:** Surface deviation evaluation results for each web surface *.

Member	Number of Points	RMSE [mm]
G1	67,948,363	3.00
G2	143,975,770	4.96
CB	13,956,745	4.90
G3	73,096,512	4.65

* G1, G2 and G3 are identifications of main girders defined in Figure 9. CB indicates the cross-beam.

## Data Availability

The point cloud data of the steel corner specimen supporting the conclusions of this article will be made available by the authors on request. The bridge point cloud data presented in this study is not readily available due to technical limitations. Requests to access the datasets should be directed to co-author, Mayuko Nishio (Email: nishio@kz.tsukuba.ac.jp).

## Abbreviations

The following abbreviations are used in this manuscript:

FE	Finite Element
PCA	Principal Component Analysis
C2M	Cloud-to-Mesh
RMSE	Root Mean Square Error
